# Informing about childbirth without increasing anxiety: a qualitative study of first-time pregnant women and partners’ perceptions and needs

**DOI:** 10.1186/s12884-023-06105-3

**Published:** 2023-11-17

**Authors:** Anne-Sylvie Diezi, Mélanie Vanetti, Marie Robert, Béatrice Schaad, David Baud, Antje Horsch

**Affiliations:** 1 Institute of Higher Education and Research in Healthcare (IUFRS), University of Lausanne, Route de La Corniche 10, 1011 Lausanne, Switzerland; 2 Communication Department, Lausanne University Hospital, Rue du Bugnon 21, 1011 Lausanne, Switzerland; 3 Institute of Humanities in Medicine, Lausanne University Hospital and University of Lausanne, Av. de Provence 82, 1007 Lausanne, Switzerland; 4 Department Woman-Mother-Child, Lausanne University Hospital and University of Lausanne, Av. Pierre-Decker 10, 1011 Lausanne, Switzerland

**Keywords:** Childbirth experience, Childbirth risks, Childbirth information, Antenatal education, Prenatal care, Shared decision making, Qualitative research

## Abstract

**Background:**

Complications requiring medical interventions during childbirth are far from rare, even after uncomplicated pregnancies. It is often a challenge for maternity healthcare professionals to know how to prepare future parents for these eventualities without causing unnecessary anxiety. Studies on traumatic birth experiences have shown that feelings of loss of control, insufficient information, and lack of participation in medical decisions during childbirth are factors of difficult experiences. However, little is known about the information and communication needs of expectant parents about childbirth during the prenatal period. To gain a deeper understanding of the information and communication needs of first-time pregnant women and partners, we explored their perceptions and expectations for their upcoming childbirth, and the actions they initiated to prepare for it.

**Methods:**

Semi-structured interviews were conducted individually with first-time pregnant women and partners of pregnant women aged 18 years or older, with an uncomplicated pregnancy. Thematic analysis was used to identify themes and sub-themes.

**Results:**

Twenty expectant parents (15 pregnant women and five partners of pregnant women) were interviewed. Six themes were identified: Childbirth event; Childbirth experience; Childbirth environment; Organisation of care; Participation in decision making; Roles within the couple and transition to parenthood.

**Conclusions:**

This study contributes to a better understanding of the information needs of future parents expecting their first child. Results highlighted that the notion of “childbirth risks” went beyond the prospect of complications during birth, but also encompassed concerns related to a feeling of loss of control over the event. Expectant parents showed an ambivalent attitude towards consulting risk information, believing it important to prepare for the unpredictability of childbirth, while avoiding information they considered too worrying. They expressed a desire to receive concrete, practical information, and needed to familiarise themselves in advance with the birth environment. Establishing a respectful relationship with the healthcare teams was also considered important. The findings suggest that information on childbirth should not be limited to the transmission of knowledge, but should primarily be based on the establishment of a relationship of trust with healthcare professionals, taking into account each person’s individual values and expectations.

## Background

A significant number of women undergo unplanned medical interventions during childbirth, often performed in an emergency, to address unexpected complications. In Switzerland, for example, nearly 16% of women face an unplanned caesarean section, 11,1% an instrumental vaginal delivery (forceps or vacuum) and 17% an episiotomy. In more than 26% of cases, childbirth is induced. In addition, many women face a serious complication, the most important being postpartum haemorrhage (8%), preeclampsia (2%) [1], and preterm birth (6.3%) [2]. The unplanned character of a medical intervention often has a negative impact on women’s birth experiences [3–5]. Prenatal communication about the labour process and the childbirth risks could better prepare future parents for these eventualities and reduce the feeling of unexpectedness. However, such a communication could also potentially reinforce the prevalence of a “risk discourse”, and transform childbirth from a natural process into a pathological one, thereby unnecessarily increasing the anxiety of couples in situations where there is no indication of complications for the upcoming birth [6, 7]. This issue seems important to take into account, as it is known that fear of childbirth is widespread among pregnant women, especially nulliparous women [8, 9].

In healthcare in general, the provision of information is widely recognised as an essential means of enabling patients to acquire the necessary knowledge to exercise autonomy and participate in decisions that affect their health [10]. The need for “informed consent” prior to any invasive medical procedure is well established and considered a fundamental right in international standards and most state laws, and is supported by a professional code of ethics and human rights organisations [11, 12]. This implies that healthcare professionals have a duty to provide sufficient information on benefits, risks, and alternatives of the medical procedure in advance, so that the patient can make informed choices. However, it has been widely noted, including in the context of maternity care, that several constraints and barriers complicate the application of informed consent in clinical practice and thus impede its effective implementation [13–15]. For clinicians, one of the central issues is to be able to manage the tension between the two fundamental ethical principles of autonomy and non-maleficence, particularly in emergency situations, where it is difficult to obtain true informed consent in the absence of a prior exchange of information with the patient [13]. In addition, to enable patient involvement in decisions, information must be adapted to their level of understanding and provide an objective and balanced perspective of possible care options, which is often not the case [16, 17]. The degree of detail of the information was also discussed, as too much information on the risks of the procedure is likely to cause a “nocebo effect”, whereby the prospect of a negative result precipitates the corresponding symptom or leads to its exacerbation [18]. These ethical dilemmas have led to a reconsideration of the process of informed consent, with greater attention to the context of care and the patient values and preferences, as well as to the relational issues between healthcare professionals and patients. Beyond informed consent, shared decision making was thus presented as a pillar of patient-centred care by ensuring patient self-determination, while building trust in the relationship between caregivers and care recipients [15, 19, 20].

In a recent international review of qualitative studies, provision of information and informed consent about labour and childbirth was presented as an essential component of respectful maternity care [21]. Several studies on satisfaction with childbirth showed that providing information and discussing the birthing process with healthcare professionals during pregnancy positively influenced women’s experience and contributed to their empowerment by giving them the opportunity to gain knowledge about medical interventions, maintain control, and participate in decision-making [22, 23]. The information seems to not only help women maintain control over what happens in their environment, but also positively influence personal control by building confidence in their own abilities [24]. Moreover, the provision of accurate and realistic information about what might happen can be a way of aligning expectations with the actual experience of childbirth and thus reducing the risk of discrepancy [25–28]. It has also been shown that preparation for childbirth through antenatal education, which integrates physical and emotional preparation in addition to the transmission of theoretical knowledge, reduces anxiety by decreasing apprehension and demystifying fears, especially for women expecting their first child [29, 30].

However, these positive findings are tempered by other studies that highlight the challenges of prenatal information and raise questions about how to best meet the expectations of future parents in the current context of maternity care organisation. Some research focusing specifically on the influence of information provision on knowledge retention, informed choice, and decision making has shown very limited results in terms of effectiveness [31–33]. The findings suggest that the context and mode of communication of information have a significant influence on its impact, and, for that to be effective, information should be coordinated and promoted by trusted care providers. In addition, it should be part of a strategy that supports future parents’ participation by inviting them to express and position themselves on possible directions, and that does not simply present the available options.

Information needs should be considered globally, taking into account all sources of information available to expectant parents, such as family and friends, but also the media and online contents [34, 35]. Future parents’ beliefs, their sensitivity to risk, and their need to engage in decision making are also essential elements to be taken into account. The approach should not be limited to the pregnant woman’s perceptions, but also include the partners who play a key role in communicating with the care teams. In other words, it seems essential to get a more nuanced and in-depth understanding of the perceptions and needs of future parents to better guide the development of information strategies on childbirth. This was precisely the purpose of this qualitative study, which aimed to explore the way in which future parents anticipate their childbirth and the actions they favour to prepare for it. By examining the issue of information from the perspective of both expectant parents, the goal was to take a comprehensive approach to their information and childbirth preparation needs, whereas studies typically focus on evaluating specific communication materials or content. In addition, we considered it important to carefully take into account parents’ expectations and concerns, as information needs, especially on issues of risk, are strongly defined by personal and subjective processes, based on individuals’ beliefs and understanding of the world [36, 37].

## Methods

A qualitative study design using semi-structured interviews was used to explore the perceptions and information needs about childbirth of future parents expecting their first child. Thematic analysis was used to identify themes and sub-themes [38].

### Participant characteristics and setting

Expectant parents, either pregnant women or partners of pregnant women, were recruited for this study. To be eligible for the study, participants had to fulfill the following inclusion criteria: (a) 21–36 weeks pregnancy, (b) no previous experience of childbirth, (c) pregnancy with no complications, (d) gave written consent. Exclusion criteria were (a) insufficient language skills to take part in a conversation in French.

In Switzerland, maternity services are covered by the compulsory health insurance. They include seven check-ups performed by a doctor or a midwife during and after the pregnancy. A unique contribution is granted to the cost of antenatal classes, whether individual or in group (CHF 150). Births at home, in the hospital or in a birthing centre benefit from the same insurance coverage. However, the vast majority of births take place in hospitals (98.3%) [1].

### Procedures

Participants were recruited on a voluntary basis through social media announcements and through advertisements within the Lausanne University Hospital and within the local federation of independent midwives. Based on the recommendations on sample size for qualitative studies, we aimed to recruit a total of 15 to 20 participants [39, 40]. As far as possible, a diversity in terms of age, migration status, education, and socio-cultural level was sought in the sample (purposive sampling). Participants were recruited on a first-come, first-serve basis, i.e., we didn’t select any participants and the recruitment stopped when the planned sample size was reached.

Eligible individuals who responded to the advertisements were contacted individually by phone to verify that they met all inclusion criteria, to explain the purpose and procedures of the study, and to answer any questions. If they were interested in the study, an appointment was scheduled for the interview. An e-mail was then sent to them with an information sheet about the study and the consent form. They were given several days to read the document and ask questions before giving their informed consent and participating in the interview.

Data were collected through individual semi-structured interviews conducted from November 2021 to March 2022 by the first author (A.D.). The first author is a communication specialist with some experience of interviewing service users. She works regularly with various clinical teams at the University Hospital and has also experienced its maternity ward as a patient. The interviews were based on an interview guide that included ten open-ended questions with follow-up questions (Table 1). The guide was developed with perinatal experts and representatives of two local parent associations. Due to COVID-19 restrictions, participants had the option of participating in the interviews via videoconference or face-to-face. Interviews were held online for a majority of participants (70%). They lasted approximately one hour and were audio recorded. In addition to the interviews, personal and socio-demographic data were collected from each participant at the beginning of the interview. All socio-demographic data were coded to protect confidentiality.

**Table 1:** Interview guide

1. What would be a normal childbirth for you?
2. When you think about your future childbirth, what are the things that are most important to you?
3. What are the situations you would not want to experience?
4. How would you describe your state of mind today at the thought of these events occurring during your childbirth?
5. What sources of information did you use to get an idea of your future childbirth?
6. How do you think your views have been influenced by these sources?
7. How important do you feel is it to prepare for the unexpected events of childbirth?
8. What steps are you taking to prepare for childbirth?
9. How important do you think is it to include information and discussion about what may happen during childbirth in prenatal care?
10. What would be the best way for you to do it?

### Data analysis

The verbatim of the interviews were transcribed and coded using a qualitative data analysis software (MAXQDA). Inductive thematic analysis method was used to analyse the data [38]. Coding was conducted by three researchers independently (A.D., communication specialist and PhD candidate; M.V. anthropologist; M.R., sociologist). The codes were then compared and discussed to extract themes and sub-themes and define the thematic map. All stages of the analysis were discussed with a senior clinical psychologist (A.H.) to ensure the credibility of the results.

## Results

Twenty expectant parents (15 pregnant women and five partners of pregnant women) were included in the study. All interviews were individual. Three of the five partners were partners of participants in the study.

At the time of the interviews, the women were between 21 and 36 weeks pregnant. Of the 20 participants, 18 were planning a hospital birth and two were planning a birth in a birth centre. Three of the 18 participants planning a hospital birth, two women and one partner, had initially chosen a birth centre but had changed their minds after visiting it, or had to give up due to the lack of availability. Six participants had already attended group or individual antenatal education classes, three were currently attending, and 11 were planning to attend in the near future. Table 2 presents the sample characteristics.

**Table 2:** Participant characteristics (*N* = 20)

	N (%)
Status	
Pregnant women	15 (75)
Partners of pregnant women	5 (25)
Duration of pregnancy (weeks)	
≤ 25	4 (20)
26–29	6 (30)
30–33	7 (35)
≥ 34	3 (15)
Antenatal education class	
Done	6 (30)
Ongoing	3 (15)
Planned	11 (55)
Age	
≤ 30	2 (10)
30–35	12 (60)
≥ 35	6 (30)
Education^a^	
Higher education (University)	8 (40)
Higher professional education	5 (25)
Secondary education and vocational training	7 (35)
Residency in Switzerland	
Native/ from early childhood	11 (55)
5–10 y	5 (25)
≤ 5 y	4 (20)

^a^Categories defined on the basis of the Swiss Federal Statistical Office’s classification of educational levels [41]

The following six themes were identified through thematic analysis: (1) Childbirth event, (2) Childbirth experience, (3) Childbirth environment, (4) Organisation of care, (5) Participation in decision making, and (6) Roles within the couple and transition to parenthood. Each theme was considered according to the two categories of questions discussed during the interviews: (a) the participant’s perceptions and expectations: what she/he expected for their upcoming birth based on her/his perception of the different concepts and situations (b) the participant’s preparation: what she/he found necessary and/or put into place to meet their expectations.

**Fig. 1:**
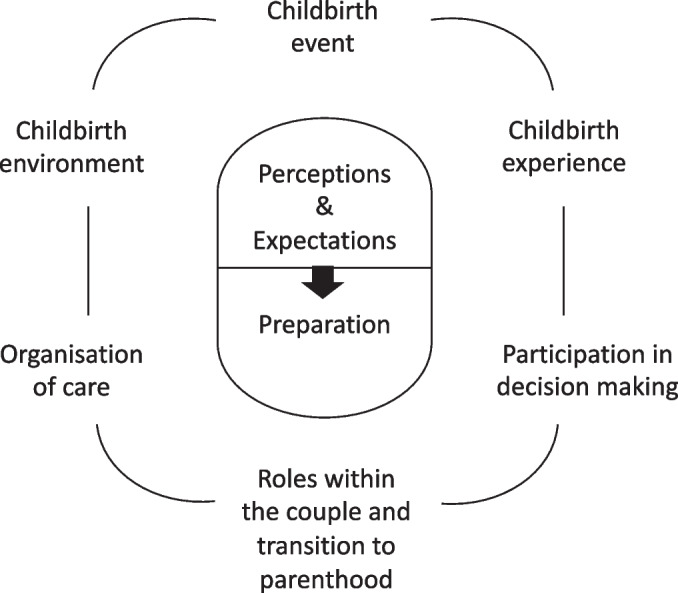
Thematic map

No important differences were found between the responses of the women and the partners, except for some specific aspects, which were detailed when necessary. For this reason, a single thematic map (Fig. 1) was developed for all participants. Table 3 offers an overview of the sub-themes for each theme.

**Table 3:** Overview of themes and sub-themes

Main themes	Sub-themes Perceptions & Expectations	Preparation
1. Childbirth event	Going natural An unpredictability to deal with No risk-taking	Practice and techniques to keep control Information (or not) on risks to prepare for difficulties
2. Childbirth experience	A unique experience to enjoy A possible sense of failure	A positive and confident frame of mind Flexibility and resilience
3. Childbirth environment	A safe environment A calm and intimate environment Personalisation of care	Becoming familiar with the environment and staff
4. Organisation of care	Reference professionals to rely on A comprehensive approach	Information for a global and concrete understanding
5. Participation in decision-making	Confidence in professional expertise A partnership to build	Communication with healthcare professionals about choices
6. Roles within the couples and transition to parenthood	As a mother, a vulnerable but leading position As a partner, a support and advocacy role	A preparation to do together Tools and knowledge to manage a new role

### Childbirth event

Participants considered childbirth to be a natural event and felt that a physiological process was the best option for mother and child. However, they were aware that childbirth was also a risky event that warranted medical intervention to some extent. In their preparation, they felt it important to acquire tools and knowledge to better control unpredictable and potentially difficult situations that could arise.

#### Perceptions and expectations

Women and partners mainly described childbirth as a natural event, for which the woman’s body was made and which generations of women had gone through without medical intervention. Some participants referred to the innate knowledge of the female body that gives the mother-to-be the confidence that she will be able to manage childbirth.

“*I believe that nature is well made, we can let it do it itself without always intervening. I trust my baby, I trust myself, and I tell myself that everything will go well, in the most natural way possible, as we were designed to do in the end.” (W2)*

In line with this perception, most participants expressed a desire for a natural birth, which they defined mainly as a vaginal birth, with or without assistance for pain management. They associated natural childbirth with fewer side effects on the health of the mother and baby, but also with a form of pride for the mother in *making it on her own* and a benefit for the baby in coming into the world *by itself*. Caesarean section was cited as the most undesirable birth option, both because of its invasive nature and because it limited the mother’s participation in the birth. One partner also expressed concern about not being able to attend the birth in the event of a caesarean section.

While guided by nature, childbirth was also considered by participants as an unpredictable event, with potential risks that could not be anticipated and over which they had little command. The uncertain nature of childbirth was presented as a stressor by some participants. They were particularly fearful of emergency medical intervention, as they associated it with potentially serious complications which, in addition to having negative consequences for the health of the mother and child, could put them out of control of the situation. Beyond the risks of the birth, they felt also uncertain about their own ability to cope with the difficulty of the event, especially in terms of duration and pain for the women. Participants said they found it hard to project themselves into the event because of this uncertainty, reinforced by the fact that they had never experienced childbirth.

“*There are lots of things I don’t know and can’t control. I don’t know when the birth will be. As soon as it starts, I don’t know how long it will last. Until I’m in the delivery room, I don’t know exactly what kind of birth it will be. For me, most of my anxiety is about many, many things that I can’t plan and that are unknown. Because it’s the first child…” (W7)*

Although all participants stated that the natural approach should definitely be preferred, they were unanimous in saying that the foremost priority in childbirth was to ensure the safety of the mother and child*.* They reported that the risks of possible complications justified medical interventions and that they had confidence in the skills of the medical team to manage these potentially dangerous situations. However, the importance given to the medical procedure varied among the participants. Some felt that childbirth is in principle a medical act, meaning that it is not simply a natural process. Others presented the option of medical intervention as a last resort after the natural process had failed, which should not, however, be questioned if the life of the mother or the child was at stake.

“*Afterwards, when it really becomes a ‘matter of life and death’, she can’t let go, it won’t open, what do we do? Well, it’s the doctors who have to take out the baby. The doctors have to cut open and everything ends well.” (P4)*

#### Preparation

Participants considered birth preparation as a way of acquiring tools that would make it easier to manage difficult situations in childbirth and thus maintain control. Women in particular expressed their need to practice exercises and techniques to help with breathing, relaxation, and pain management.

*“For example, prenatal yoga, I think it will also help me a lot to breathe to relax. And to take positions that also relieve the contractions, the pain, and also positions that can help the baby to descend. So, I think that’s very important. And it’s reassuring, too”. (W13)*

Most women and partners shared the idea that knowing about possible complications and medical interventions was a way of preparing for difficulties, anticipating them, and thus accepting them more easily if they occurred. However, the need for information and the active search for content varied among the participants. Some participants reported that information was a way to manage the uncertainty of the event, as it gave them the tools to understand the situation and to know that solutions existed. Others preferred, instead, to protect themselves from potentially frightening content, and consciously decided to limit their search for information. Such ambiguous relationship to information was spontaneously expressed by some participants, who did not know what was best for them in the end.

*“So, I think it’s maybe more my nature to avoid looking for information too much. Even though I also need to know. It’s quite paradoxical.” (W10)*

### Childbirth experience

Childbirth was described by women and partners as a special experience, different for everyone. Some women expressed the challenges of a *successful* birth and the sense of failure they might feel if it did not go as planned, suggesting that childbirth had an impact on their lives beyond the event itself. Considering that the mindset played an important role, a majority of participants reported favouring a positive and resilient attitude in their anticipation of birth.

#### Perceptions and expectations

Although risky and difficult, childbirth was described by several participants as a *unique* and *magical* moment that could only be truly understood by living it personally. Some women expressed curiosity and excitement about the experience of giving birth, which they perceived as a privilege, to the point of wishing that the birth would last long enough to be fully enjoyed. These women underlined the importance to be actively engaged in their childbirth, which became a kind of existential experience.

*“To live the intensity of something. And then actually to experience something that lasts a little bit. I think that if it lasts 30 minutes or an hour, it would not be enough. Because I have the impression that it is still a transition and then I want to live it, to seize it.” (W14)*

While most women said that they would do everything possible to promote a natural birth, sometimes seeing it as a form of challenge and a source of personal pride, several expressed to be very aware of the social pressure they were under, as a successful birth should be vaginal, ideally without an epidural. These women mainly reported protecting themselves from such injunctions, which they saw as harmful. However, two women expressed the sense of disappointment that could result from a birth that would not go as planned. The worst for them was not to face difficulties in the context of the birth, but to have to live with a sense of failure afterwards.

*“If I arrive at the maternity hospital and they tell me ‘Mrs. X., you have to do a C-section, there is no choice’, I think that it is not at that moment that it hurts me the most or that it scares me the most […]. It’s more afterwards, I’m afraid to say to myself, that I didn’t have the birth I wanted.” (W1)*

#### Preparation

While they knew that childbirth could be a difficult experience, most women and partners indicated that they wanted to remain in a positive and confident state of mind when considering their upcoming birth. Some were well aware that this was based on a form of positive self-suggestion, which allowed them to approach childbirth with more serenity. This optimism seemed all the more possible, as the pregnancy had gone well up to that point and they had confidence in the competence of the professionals, in nature, or in their own ability to cope with the situation, whatever it might be.

*“I would say confident. Reassuring. But I have the feeling that everything will go well. My state of mind is ‘everything will be fine’”. (P3)*

In addition to this positive mindset, most participants confided that they were prepared to be flexible and resilient, and therefore open to any situation, however difficult, that went against what they had hoped for. They reported that flexibility was necessary due to the unpredictable nature of childbirth and that the most important point, in the end, was that the mother and the baby were healthy. Some participants also expressed their reluctance to over-plan the event, to avoid frustration if their childbirth did not go according to expectations. In this respect, birth plans were sometimes perceived negatively because they gave the illusion that the birth could be planned in advance.

*“I say to myself, the more I plan, the more I say to myself that this is what I want, the more in my opinion I have the impression that we’re going to get out of this theoretical plan because, I don’t know, a lot of things can happen, and if I plan too much, it’s going to frustrate me even more.” (W6)*

### Childbirth environment

Safety was perceived by women and partners as an essential criterion of the childbirth environment, which explained their choice for a hospital birth. At the same time, participants expressed the importance of having an attentive, respectful, and personalised environment, both in their relations with professionals and in the layout of the birthing facilities.

#### Perceptions and expectations

In line with their desire not to take risks, the majority of participants underlined the importance of choosing a place of birth that could guarantee the safety, both in terms of the equipment available and the qualifications of professionals. The participants confided that the choice of a hospital birth was mainly motivated by their need for security. The preference for a birth centre, when expressed, was only acceptable because of its proximity to a hospital. The university hospital, in particular, was seen as a highly reassuring place because *everything was there* in case of complications.

*“That there are instruments around, instruments that I don’t really know what they’re for, but it reassures me to have lots of high-tech instruments around me, saying to myself, we don’t know, but they’re there.” (P1)*

Women in particular reported their need to give birth in a calm environment. The hospital was perceived as a potentially stressful place, due to the heavy workload for healthcare professionals and the emergencies that had to be managed. Several women expressed concern about the presence of a large number of professionals during their birth, because of the noise and bustle, but also because of the lack of privacy that this presence could bring.

*“And then it’s true that I’m quite shy, so I’m also a bit afraid of finding myself with several people in front of me and not being at ease, of being inhibited, in fact, in relation to that.” (W8)*

The benevolence and kindness of the midwifery staff, as well as their ability to listen and to personalise care, were regularly mentioned by women and partners as important criteria for a positive experience of childbirth. Participants hoped that the teams would be welcoming and respectful, but also that the facilities could be adapted to their wishes. Hospital was a priori not perceived as a very flexible and hospitable environment. Through their comments, participants expressed their concerns about experiencing a form of dehumanisation and their need to be considered as people beyond the care of the body.

*“… that they respect the fact that we are... I mean, we’re naked here, that they have respect for our bodies and that we’re not just women giving birth. That we are still a person.” (W13)*

#### Preparation

In their preparation to childbirth, women and partners expressed the need to become familiar with the environment of their upcoming birth, although they were aware that this was not always possible. Depending on the individual, this need was expressed in different ways. Some participants felt it was important to get information about the professionals who would be present, and ideally to be able to meet them beforehand. For others, it was the visit to the birthing place in advance that was essential. Two women also reported that they had prepared a music playlist with their partner for the day of the birth. These different approaches were presented by the participants as ways of anticipating and mastering the context of an unknown and potentially difficult event.

*“Presenting the places can help, the rooms. It’s like when you take an exam. I was always more comfortable when I took an exam in a room I knew.” (W9)*

### Organisation of care

Participants observed a discontinuity in the organisation of maternity care and regretted not having the same reference person throughout their preparation and experience of childbirth. Discontinuity was also observed in terms of information, which participants perceived as insufficient and too fragmented to meet their needs in preparing for birth.

#### Perceptions and expectations

In their answers, some participants mentioned their difficulty in identifying reference professionals for questions related to the birthing process. Several women in particular indicated that their private gynaecologist was their primary trusted contact during pregnancy, but did not talk to them about the birthing phase, as they would not be involved. Midwives were seen as the professionals responsible for managing childbirth, and therefore essential in the preparation phase, but those encountered before childbirth would not, in principle, be those who would ensure the birth. In addition, the hospital was perceived as a complex, potentially overburdened system, with many professionals involved and regular turnover. For some, this discontinuity in the organisation of care added uncertainty to an already unpredictable event. In this context, being able to identify a reference person, at least on arrival at the hospital, was seen as a source of reassurance.

*“I think it would be nice (...) to have a person who is present from the beginning to the end. For example, that it is the same midwife who welcomes me when I arrive at the maternity hospital and who follows the whole birth process, and that even if there’s a problem or something that wasn’t foreseen, that this person is always present and gives me the information.” (W5)*

In terms of their understanding of care, some women expressed regrets that there was a lack of information on certain topics related to the normal course of childbirth, while much of the content focused on complications and medical interventions to manage them. They were surprised, for example, not to find explanations about the delivery of the placenta and the standard issues related to the postpartum period, such as heavy blood loss, the pain of breastfeeding, and the psychological distress that could be felt after the birth. They observed that professionals did not address these topics, but that information was equally difficult to obtain from their friends or relatives, as if such subjects were taboo. In addition, some women, particularly those who had recently arrived in Switzerland, expressed confusion about the organisation of maternity care and reported a lack of information about the available resources, health insurance coverage, and the administrative steps to be taken for the birth of their child. They suggested that a comprehensive, centralised approach to information needs on all aspects of birth preparation would be a great help, as concerns of future parents were extremely diverse at this stage of life.

*“It is a whole thing actually. There is the pregnancy, the preparation for the baby, the administration, and then the childbirth. In fact, there are many things.” (W4)*

#### Preparation

Most participants indicated that they had sought or were seeking information about the different stages of childbirth to better understand its usual course. They were particularly eager for practical and realistic information that would allow them to anticipate their upcoming birth, to put it into context, and to better understand the care that would be provided to the mother and child. They perceived the birth preparation given by midwives as particularly valuable, especially when it was provided by their birthing facility because it conveyed information that was consistent with the reality they would experience. Some participants said they favoured feedback from family and friends because they were about real experiences. Several women and partners also mentioned watching information videos online, which gave them a more concrete and visual understanding of birth.

*“I watch some videos too, on Youtube (...) I’m interested to know exactly what happens because it’s true that when someone tells me about their childbirth, it’s never in detail of what exactly happens.” (W12)*

### Participation in decision-making

Women and partners reported high confidence in the knowledge and experience of healthcare professionals and did not plan to question their decisions. However, they hoped to be respected in their expectations and to receive explanations about the medical procedures performed during childbirth. They confided that their latitude of choice in decision-making was unclear and needed to be clarified before birth.

#### Perceptions and expectations

In general, participants reported a high level of trust in healthcare professionals, obstetricians, and midwives, whom they felt were in a better position to make decisions because of their knowledge and experience. For this reason, they did not consider questioning medical decisions, as long as such decisions were justified and not made for the comfort of the healthcare team. For some participants, going against medical choices could disrupt the system and affect the quality of care, or even endanger the mother and the child, which they wanted to avoid at all costs.

*“I have no desire to impose things that will ultimately derail the system, in fact. That it would be too complicated for them to take charge and (...) that it would be a bit badly done in the end. That it could be harmful, when it was perhaps just an idea, a whim.” (W9)*

Although they recognised that their involvement was limited because of the asymmetry in knowledge and experience, participants felt it was essential that some form of partnership took place around birthing decisions. They expected the teams to maintain communication with them throughout the birth and to consider them as full partners in care. It was especially important for them to be informed of any medical intervention performed and to know the reason for it. Indeed, even if they did not feel they had decision-making power, they expected the teams to justify the medical decisions, so that they could understand and accept them. It was for them a way to remain involved in the birth, to keep control, and to better accept the difficulties that may arise.

In order to build trust with the teams, it was equally important to the participants that the preferences they had expressed were heard and respected. They hoped that the teams would accompany and support them in the way they wanted to foster. Some women, in particular, feared that they would be pressured by healthcare professionals to accept medical interventions to make the childbirth go faster.

*“[I expect] to be heard in my questions, in my doubts, that they answer me if I have questions during the birth. That they respect the natural or biological rhythm, that they won’t want to accelerate the process.” (W11)*

#### Preparation

Most women and partners expressed uncertainty about the degree of control they had over decisions about the birthing process. They therefore hoped to be able to discuss their preferences in advance with a professional at the maternity hospital where they planned to give birth. Although most of them were prepared for a potential need to re-specify their expectations to the team on the day of birth, they stated that such a discussion would be a way for them to verify that their wishes were acceptable, and to be able to anticipate with their partner the choices they might have to reconsider.

*“Meeting the midwife beforehand, it’s more the idea that there is someone who knows. That the day I arrive, I don’t need to negotiate, that I know in fact that all that is possible, that I know how it is received. Because in reality (...) how do we make sure that everyone is on the same wavelength? Or even if it’s a possibility to be on the same wavelength?” (W15)*

### Roles within the couple and transition to parenthood

Participants agreed that women had a central role in childbirth but that partner involvement was essential as a support and interlocutor with the healthcare team. It was necessary for them, and especially for the women, to be involved together in the preparation for the birth of their child. This preparation went beyond the birth event itself and also involved facing their new responsibilities as parents.

#### Perceptions and expectations

Women and partners agreed that mothers played a central role in the birthing process and therefore had priority within the couple for decision-making, even though their participation might be limited because of their vulnerability during childbirth. Their leading position was justified by the fact that their own body was at stake, and that they themselves were enduring the potential difficulties associated with the birthing experience, particularly pain.

*“In 99% of cases, it is the mother who has to give her opinion. I just give my opinion but it is not me who chooses, it is not my body.” (P2)*

Although they sometimes felt they had a very limited role in the birth, partners reported being very concerned about providing support and reassuring the mother during the birth, so that she could have the best possible experience. Some noted the limitations in their support role, due to the fact that they themselves would be emotionally involved, and that the situation might not allow them to meet all their partner’s expectations. For their part, all the women interviewed stressed the importance of having their partner by their side during childbirth. They confided that this presence would give them indispensable support and also a form of security in the painful experience they might have to endure. In addition, several women reported that they were very reassured that their partner could be their advocate with the team in case they were no longer able to express their preferences.

*“And even if I were to be in a vulnerable position, I know that he would be there to be my voice if I wanted him to. I feel like it really takes a lot of stress out of dealing with this situation that’s probably going to be stressful, that’s not going to be the best time in life.” (W15)*

#### Preparation

Participants indicated that it was important for them to be able to share their birth preferences with their partner before the birth, so that they would be in agreement for their discussions with healthcare teams. At the same time, several women reported that they felt their partner was not enough involved in childbirth preparation and that they were considering different approaches to get him to access the necessary information, such as sharing information resources with him or attending antenatal classes together.

*“The antenatal class, for example, I’m going to attend it especially so that my husband feels involved because, for the moment, every time he talks about it, he’s a spectator. I tell him that you can be a little more involved, so I tell myself that maybe this will allow him to see childbirth differently.” (W2)*

Several participants were particularly concerned about preparing for their future role as parents. This preparation had different objectives. It focused in part on practical issues, particularly related to the choices to be made for the purchase of equipment for the baby. It also dealt with more psychological considerations related to their new responsibilities as parents and the balance to be found within the couple. The partners, in particular, said that it was important for them to prepare for their role as fathers and to make the arrival of their child more tangible by beginning to establish a bond with him/her during the pregnancy already, using techniques, such as haptonomy or communication through singing or speaking.

*“But it’s something that I have to prepare, prepare for the arrival of the child in advance, that is to say to be ready for the arrival, to have a baby in the womb but to humanise it in advance.” (P5)*

## Discussion

The aim of our study was to explore the way in which first-time pregnant women and partners anticipated the birthing process and the actions they favoured to prepare for it. The qualitative approach was chosen to gain a more in-depth and comprehensive understanding of their information and communication needs in order to guide future communication strategies. The thematic analysis of the interviews allowed us to identify six themes that characterised the different areas of concern of future parents facing a first birth: Childbirth event; Childbirth experience; Childbirth environment; Organisation of care; Participation in decision making; Roles within the couple and transition to parenthood.

### To know or not to know: an ambivalent position towards risk information

In our study, pregnant women and partners described childbirth as an unpredictable event that could potentially require medical intervention to manage complications. Even those who were planning an out-of-hospital birth considered safety to be a key criterion in their decision making. These views are not surprising in a country like Switzerland where the medicalised model of childbirth care predominates, with 98.3% of births taking place in hospitals [1]. At the same time, all expectant parents defined birth as a natural event, and expressed a preference for a physiological approach, which they perceive as the “normal” course of childbirth. Consistent with other research on childbirth expectations [42, 43], the participants thus combined a priori opposing visions: childbirth was a potentially risky process, but one that should happen naturally; it was a difficult moment to go through, but at the same time a unique experience to enjoy. These findings support the approach advocated by several authors that suggested that the polarised division between natural and medicalised birth is no longer relevant and that these concepts should be considered on a continuum rather than in opposition to each other [44–46]. Although individual values and beliefs led participants to tend more to one side or the other, the expectations and preparation for childbirth appeared to us as an attempt by expectant parents to articulate and reconcile these two perspectives to find a reassuring space in between.

This ambivalent attitude seems to influence how expectant parents seek and deal with information about childbirth risks. Most participants considered it important to be informed about possible complications, such knowledge allowing them to anticipate adverse events and to get involved in decisions. At the same time, however, they tried to avoid this type of information, preferring an optimistic or resilient attitude, sometimes suggesting that a negative state of mind could adversely influence the course of events. Research has shown that people are not passive recipients of risk information and that they do not respond to it rationally [47]. They can be active in seeking and using information, but can also make conscious decisions to avoid certain topics that they do not consider relevant to their needs. Excessive optimism in the face of possible negative events has also been described as an adaptive ability to reduce the stress and anxiety at the prospect of difficult experiences [48]. But more specifically, the attitudes of the expectant parents seem to be related to what Levy defined as the desire to “maintain equilibrium” in her study on women’s informed choices during pregnancy [49]. She described how pregnant women regulated information, avoiding it if it did not allow them to change the situation or if it was potentially disruptive because of its stressful nature. In line with this idea, it seems to us that the participants in our study chose to avoid information about possible childbirth complications because they felt it was not helpful in preventing adverse events, while potentially jeopardizing their positive expectations about their upcoming birth. However, even if participants chose to avoid the information on possible complications, most of them still highlighted the importance of this information being reliably available.

### Preparing for the “reality” of childbirth

A mismatch between expectations and experiences of childbirth has been found to have a negative impact on women’s satisfaction and their postpartum mental health [26, 50, 51]. For this reason, several authors have emphasized the importance of giving pregnant women a more realistic picture of childbirth, especially for first-time mothers who have no previous experience and who may develop “romanticised” expectations [52]. To address this need, prenatal information and educational programs have been considered as a way to enhance the knowledge of expectant parents and thus prepare them for the realities of the childbirth experience [25, 27, 53]. The results of our study do not seem to support this approach. Indeed, although facing their first childbirth, the participants did not show particularly idealised or unrealistic expectations about their upcoming birth. On the contrary, they seemed to be well aware of its unpredictability and the possibility of complications that could go against their wishes. Thus, even if they hoped that the birth would go smoothly and naturally, in accordance with their values and desires, this did not mean that they were particularly lacking in “knowledge” about possible childbirth adverse events. This nuance is of importance and suggests that preparation to the risks of childbirth should go beyond the simple transmission of information content to prepare future parents for a new and particularly unpredictable experience.

In addition, while participants expressed concerns about the prospect of a complication during childbirth, it appeared that beyond the event itself, they were particularly preoccupied with its impact on their own experience. The participants’ main concern was to ensure the health of the child and the mother, but they reported trusting the expertise of professionals to manage risky situations that could potentially threaten it. However, they were preoccupied that such adverse events would make them feel out of control of the situation. In addition, many pregnant women expressed worries that they would not be able to cope with the pain and duration of labour and therefore not feel fully involved in their childbirth. For some, it seemed that the risk of being somehow dispossessed of their birth could have consequences beyond the event itself, and give them a sense of failure that they would have to live with.

Participants’ concerns are consistent with several studies that have shown that feeling in control during childbirth is a significant predictor of a positive childbirth experience [22, 54–57]. As described in the literature, control can be of two different kinds: it can be either internal (referring to the woman’s ability to control her feelings and body) or external (referring to the parents’ relationship with their environment and their ability to participate in decisions) [58]. It is interesting to note that in their preparation for childbirth, participants were looking for ways to strengthen both types of control. Women in particular emphasised the importance of acquiring tools, such as relaxation or breathing techniques, expressing a need for practice in their preparation for childbirth in order to develop coping strategies [59]. Pregnant women and partners also reported a desire to clarify their role in decision-making during childbirth, as their degree of control was unclear to them. Their need to visit the place of birth and meet the staff beforehand also appears to us, in some ways, as a way of reinforcing their control in anticipation of their upcoming birth.

### A need for a contextualised and personalised information

In addition to familiarising themselves in advance with the birth environment, expectant parents wished to receive more concrete and practical information about the natural birthing process, as well as a more complete understanding of the services available to them. While antenatal classes and testimonials from family members or friends were particularly valued, it is interesting to note that these means were not felt to be sufficient, and that searching for information on the internet, particularly videos, was often cited as a useful way to visualise and anticipate concretely what they were about to live.

The fact that the participants in our study were experiencing their first pregnancy may explain this strong need for concrete, practical information [53]. However, their comments also seem to reflect the impact of fragmentation and specialisation of maternity care on their communication needs. Indeed, the multiplicity of providers is less conducive to a holistic consideration of the full range of physical, emotional, and organisational issues in the transition to parenthood. Furthermore, by fragmenting information about pregnancy and childbirth, focusing on very specific and specialised information, there is a risk of neglecting the very pragmatic needs of expectant parents, either because they are considered to be outside the scope of concerns and responsibilities, or because they are seen as so common that they are trivialised. These findings argue in favour of developing continuity-of-care models in maternity care to enhance the quality of information provision.

Several studies on antenatal information and education have highlighted the importance of interaction with healthcare professionals and support to contextualise information, address personal concerns, and manage the emotional aspects of the birth experience [3, 60–62]. Participants in this study expressed a similar wish for personalised care, respect and empathy from healthcare teams, which seemed to be all the more important, as the hospital was perceived as a particularly inhospitable and inflexible environment. This need, also expressed by patients in other care settings, can be interpreted as a desire to be recognised as persons, with specific and individual needs, in a contemporary care context that tends to dehumanise the care relationship [63].

### Building trust

Today, more than ever, expectant parents have access to a multitude of information resources on childbirth. Contrary to what one might think, this reality does not seem to undermine confidence in the expertise of healthcare professionals, who are still considered as the most valued sources of information [31, 61]. In our study, pregnant women and partners confided that they did not want to question the medical decisions made by professionals, thereby attesting to their faith in the medical approach to ensure a safe birth. Some participants even feared that their involvement in decisions could compromise the outcome of the birth by negatively influencing its course.

This does not mean that future parents planned to remain passive, but the partnership they expected seems to be characterised more by the quality of the relationship on which it would be built than by the decisions it would involve. Participants expressed a desire to clarify in advance how much of the decision making they would be involved in regarding birth-related choices, and whether their wishes were compatible with the usual procedures of the birthplace. Rather than unilaterally communicating their wish list or birth plan, it seems that their need was primarily to engage in a dialogue in order to adjust their own perspectives to the birthing context that would offer them the expected safety. We interpret this as a desire to initiate a relationship of trust with the professionals who will take care of them, thus preparing a model of partnership that would allow them to be involved in decision-making during the birth process, seeing their wishes respected and being informed to understand what is happening, without having to carry all the responsibility for decisions. From this perspective, the expectations of future parents seem to support the argument that the quality of the care relationship is an essential foundation for successful and collaborative shared decision-making [15, 20, 64].

When considering how best to implement shared decision making in maternity care, it is essential to pay particular attention to the specific role and needs of the partners. In our study, we did not notice any major differences in the perceptions of future mothers and partners, which led us to believe that the variability of views was more related to individual characteristics than to gender criteria. However, some differences emerged in how they each anticipated the partner’s involvement in the birthing process. Although all shared the idea that the partner was a crucial support and advocate for the birthing woman, partners were apprehensive about the weight of this responsibility when they themselves would be emotionally involved in the event. In addition, they had difficulty imagining concretely the role they might play, an insecurity that was shown to persist during the birthing process and postnatal period [65, 66]. On the other hand, several women reported that they sought to involve their partners more actively in the childbirth preparation, confirming the high expectations they had of them to cope with the childbirth experience. Such discrepancies in women’s and partners’ concerns and expectations are likely to generate tensions and should therefore be further explored to ensure that prenatal communication meets the needs of each expectant parent.

## Strenghts and limitations

This study has several strengths. It addresses the question of information needs regarding the risks of childbirth in an innovative way by exploring them through the perceptions of expectant parents and the actions they take to prepare for childbirth. The qualitative approach allowed for a deeper and more subtle understanding of the needs of first-time parents through the nuances they provided during the interviews. The study also has the value of including partners, who have long been overlooked in studies addressing birth satisfaction. Finally, by focusing on future parents with uncomplicated pregnancies, our research also addresses new insights into the issue of risk information in non-pathological situations, an area in which knowledge is still very limited.

The limitations of the study are primarily related to the lack of sociodemographic diversity among the participants. Proportionally, the sample had a higher level of education than the general population, which may have an impact on how information is sought and managed, and how relationships with care teams are viewed. The lack of cultural diversity is also a limit and future research should certainly involve more people with a migration background. In addition, although we tried to include as many partners as pregnant women, the latter participated in greater numbers. The difficulty of recruiting partners in perinatal studies has already been highlighted by other authors [66]. However, this seems to have had a limited impact on the findings of the study, as we did not find substantial differences between the perspectives of women and partners, suggesting that perceptions and expectations are more related to individual differences than to gender.

## Implications for practice and suggestions for future research

Our results provide interesting insights for the development of information and communication strategies on childbirth for parents expecting their first child. First, they highlight the importance of redefining and broadening the concept of “childbirth risks” to take greater account of parental concerns. As the worries of future parents seem to be strongly linked to the fear of a loss of control, the question arises as to how prenatal information and communication could prepare them to participate more actively in the birth process and in decision making. Efforts should be made to provide pregnant women and partners with more concrete and comprehensive information to help them project themselves more easily into the upcoming birth, and to better manage the emotional issues associated with this unknown event. Visits to birthing facilities before the birth, presentations of the care teams and the organisation of care on site or via online video, or any other means enabling couples to familiarise themselves with the birthing environment in advance are also to be encouraged.

But beyond the issue of the topics to be transmitted to future parents, the question of *how* to communicate them seems essential. Our results suggest that the challenge is not simply to pass on knowledge to future parents to better prepare them for the reality of childbirth, but that this knowledge has to be contextualised and put into perspective with individual needs in order to make sense of it. The participants wished to be able to discuss their views and preferences with the team before the birth, in order to adjust their perspectives and establish a form of partnership adapted to the realities of the care environment. This prenatal exchange would seem to us to be an opportunity to initiate a collaboration between couples and healthcare teams before the birth. The aim would not be to pitch the “medical” against the “natural”, but rather to foster a flexible state of mind and mutual trust in anticipation of the birth to come, while respecting the constraints and values of each party.

To determine whether parents’ perception of their information needs changes after childbirth experience, it would be interesting to re-interview the same participants who have since given birth. Future research is also needed to compare the perceptions and expectations of future parents with those of healthcare professionals, in order to detect any discrepancies that could hinder communication. Finally, it would be useful to identify more concretely the elements to be considered in prenatal communication to encourage the establishment of a relationship of trust and support shared decision-making that meets the needs of everyone—women, partners, and professionals. In this respect, specific efforts should also be made to assess how to concretely tailor information provision and discussion on the individual needs of each future parent. To this end, we consider that it would be essential to involve all stakeholders—parents and professionals—in these research processes, in a co-development approach.

## Conclusion

This study contributes to a better understanding of the information needs of future parents expecting their first child. Results highlighted that the notion of “childbirth risks” goes beyond the prospect of complications during birth, but also encompasses concerns related to a feeling of loss of control over the event. Expectant parents showed an ambivalent attitude towards consulting risk information, believing it important to prepare for the unpredictability of childbirth, while avoiding information they considered too worrying. They expressed a desire to receive concrete, practical information, and a need to familiarise themselves in advance with the environment of their future birth. Establishing a respectful and caring relationship with the healthcare teams before labour starts was also seen as important to prepare for birth. The findings suggest that information on childbirth should not be limited to the transmission of knowledge, but should primarily be based on the establishment of a relationship of trust with healthcare professionals, taking into account each person’s individual values and expectations.

## Data Availability

In order to protect the privacy of participants, the individual data set generated in this study is not publicly available. However, the final data set for data analysis is available from the corresponding author upon reasonable request.

## References

[1] Statistique médicale des hôpitaux. Accouchements et santé maternelle en 2017. Bundesamt für Statistik (BFS); Mai 2019. https://dam-api.bfs.admin.ch/hub/api/dam/assets/8369419/master.

[2] Mortalité infantile et santé des nouveau-nés, en 2022. Bundesamt für Statistik (BFS). 2023. https://dam-api.bfs.admin.ch/hub/api/dam/assets/28005917/master.

[3] Handelzalts JE, Waldman Peyser A, Krissi H, Levy S, Wiznitzer A, Peled Y (2017). Indications for emergency intervention, mode of delivery, and the childbirth experience. PLoS One.

[4] Blomquist JL, Quiroz LH, Macmillan D, McCullough A, Handa VL (2011). Mothers’ satisfaction with planned vaginal and planned cesarean birth. Am J Perinatol.

[5] Waldenström U, Hildingsson I, Rubertsson C, Rådestad I (2004). A negative birth experience: prevalence and risk factors in a national sample. Birth.

[6] Scamell M, Alaszewski A (2012). Fateful moments and the categorisation of risk: Midwifery practice and the ever-narrowing window of normality during childbirth. Health Risk Soc.

[7] Bisits A (2016). Risk in obstetrics – perspectives and reflections. Midwifery.

[8] O'Connell MA, Leahy-Warren P, Kenny LC, O'Neill SM, Khashan AS (2019). The prevalence and risk factors of fear of childbirth among pregnant women: a cross-sectional study in Ireland. Acta Obstet Gynecol Scand.

[9] Shakarami A, Mirghafourvand M, Abdolalipour S, Jafarabadi MA, Iravani M (2021). Comparison of fear, anxiety and self-efficacy of childbirth among primiparous and multiparous women. BMC Pregnancy Childbirth.

[10] Cecchi C (2008). La place de l'information dans la décision en santé publique. Santé Publique.

[11] Académie Suisse des Sciences Médicales / Fédération des médecins suisses. Bases juridiques pour le quotidien du médecin. Un guide pratique. 2022. 10.5281/zenodo.7148478.

[12] United Nations Educational SaCO (2005). Universal Declaration of Human Rights. Records of the 33rd session of the general conference Paris.

[13] Pierre F (2018). Information de la femme et consentement en obstétrique. RPC Prévention et protection périnéale en obstétrique CNGOF. Gynécol Obstét Fertil Sénol.

[14] Nicholls J, David AL, Iskaros J, Lanceley A (2022). Patient-centred consent in women’s health: does it really work in antenatal and intra-partum care?. BMC Pregnancy Childbirth.

[15] Begley K, Daly D, Panda S, Begley C (2019). Shared decision-making in maternity care: acknowledging and overcoming epistemic defeaters. J Eval Clin Pract.

[16] Coulter A, Entwistle V, Gilbert D (1999). Sharing decisions with patients: is the information good enough?. BMJ.

[17] Delotte J, Schumacker-Blay C, Bafghi A, Lehmann P, Bongain A (2007). Medical information and patients’ choices. Influences on term singleton breech deliveries. Gynecol Obstet Fertil.

[18] Cohen S (2014). The nocebo effect of informed consent. Bioethics.

[19] Barry MJ, Edgman-Levitan S (2012). Shared decision making–pinnacle of patient-centered care. N Engl J Med.

[20] Elwyn G, Frosch D, Thomson R, Joseph-Williams N, Lloyd A, Kinnersley P (2012). Shared decision making: a model for clinical practice. J Gen Intern Med.

[21] Shakibazadeh E, Namadian M, Bohren MA, Vogel JP, Rashidian A, Nogueira Pileggi V (2018). Respectful care during childbirth in health facilities globally: a qualitative evidence synthesis. BJOG.

[22] Gibbins J, Thomson AM (2001). Women’s expectations and experiences of childbirth. Midwifery.

[23] Preis H, Lobel M, Benyamini Y (2018). Between expectancy and experience: testing a model of childbirth satisfaction. Psychol Women Q.

[24] Franzon ACA, Oliveira-Ciabati L, Bonifácio LP, Vieira EM, Andrade MS, Sanchez JAC (2019). A communication and information strategy in health and preparation for childbirth: a randomized cluster trial (PRENACEL). Cad Saude Publica.

[25] Lally JE, Murtagh MJ, Macphail S, Thomson R (2008). More in hope than expectation: a systematic review of women’s expectations and experience of pain relief in labour. BMC Med.

[26] Kahalon R, Yanushevsky Cnaani G, Preis H, Benyamini Y (2022). The complex effects of maternal expectations on postpartum depressive symptoms: when does a protective factor become a risk factor?. J Psychosom Obstet Gynecol.

[27] Churchill AC, Davis CG (2010). Realistic orientation and the transition to motherhood. J Soc Clin Psychol.

[28] Crossley M (2007). Childbirth, complications and the illusion of ‘choice’: a case study. Fem Psychol.

[29] Karabulut Ö, CoşkunerPotur D, Doğan Merih Y, CebeciMutlu S, Demirci N (2016). Does antenatal education reduce fear of childbirth?. Int Nurs Rev.

[30] Kızılırmak A, Başer M (2016). The effect of education given to primigravida women on fear of childbirth. Appl Nurs Res.

[31] Stapleton H, Kirkham M, Thomas G (2002). Qualitative study of evidence based leaflets in maternity care. BMJ.

[32] Brinkler R, Edwards Z, Abid S, Oliver CM, Lo Q, Stewart A (2019). A survey of antenatal and peripartum provision of information on analgesia and anaesthesia. Anaesthesia.

[33] O'Cathain A, Walters SJ, Nicholl JP, Thomas KJ, Kirkham M (2002). Use of evidence based leaflets to promote informed choice in maternity care: randomised controlled trial in everyday practice. BMJ.

[34] Grimes HA, Forster DA, Newton MS (2014). Sources of information used by women during pregnancy to meet their information needs. Midwifery.

[35] Sanders RA, Crozier K (2018). How do informal information sources influence women’s decision-making for birth? A meta-synthesis of qualitative studies. BMC Pregnancy Childbirth.

[36] Horlick-Jones T (2005). Informal logics of risk: contingency and modes of practical reasoning. J Risk Res.

[37] Renn O (2010). The contribution of different types of knowledge towards understanding, sharing and communication risk concepts. Catalan J Commun Cult Stud.

[38] Braun V, Clarke V (2006). Using thematic analysis in psychology. Qual Res Psychol.

[39] Malterud K, Siersma VD, Guassora AD (2016). Sample size in qualitative interview studies: guided by information power. Qual Health Res.

[40] Moser A, Korstjens I (2018). Series: practical guidance to qualitative research. Part 3: sampling, data collection and analysis. Eur J Gen Pract.

[41] Education Statistics 2022. Federal Statistical Office. Mai 2023. https://dam-api.bfs.admin.ch/hub/api/dam/assets/24586340/master.

[42] Downe S, Finlayson K, Oladapo OT, Bonet M, Gülmezoglu AM (2018). What matters to women during childbirth: a systematic qualitative review. PLoS One.

[43] Fenwick J, Hauck Y, Downie J, Butt J (2005). The childbirth expectations of a self-selected cohort of Western Australian women. Midwifery.

[44] Coxon K, Scamell M, Alaszewski A (2012). Risk, pregnancy and childbirth: what do we currently know and what do we need to know? An editorial. Health Risk Soc.

[45] Walsh DJ (2010). Childbirth embodiment: problematic aspects of current understandings. Sociol Health Illn.

[46] Brubaker SJ, Dillaway HE (2009). Medicalization, natural childbirth and birthing experiences. Sociol Compass.

[47] Alaszewski A, Horlick-Jones T (2003). How can doctors communicate information about risk more effectively?. BMJ.

[48] Sharot T, Korn CW, Dolan RJ (2011). How unrealistic optimism is maintained in the face of reality. Nat Neurosci.

[49] Levy V (1999). Maintaining equilibrium: a grounded theory study of the processes involved when women make informed choices during pregnancy. Midwifery.

[50] Mei JY, Afshar Y, Gregory KD, Kilpatrick SJ, Esakoff TF (2016). Birth plans: what matters for birth experience satisfaction. Birth.

[51] Webb R, Ayers S, Bogaerts A, Jeličić L, Pawlicka P, Van Haeken S (2021). When birth is not as expected: a systematic review of the impact of a mismatch between expectations and experiences. BMC Pregnancy Childbirth.

[52] Beaton J, Gupton A (1990). Childbirth expectations: a qualitative analysis. Midwifery.

[53] Dahlen HG, Barclay L, Homer CSE (2010). ‘Reacting to the unknown’: experiencing the first birth at home or in hospital in Australia. Midwifery.

[54] Meyer S (2013). Control in childbirth: a concept analysis and synthesis. J Adv Nurs.

[55] Melender HL (2002). Experiences of fears associated with pregnancy and childbirth: a study of 329 pregnant women. Birth.

[56] Hodnett ED (2002). Pain and women’s satisfaction with the experience of childbirth: a systematic review. Am J Obstet Gynecol.

[57] Fair C, Morrison T (2011). The relationship between prenatal control, expectations, experienced control, and birth satisfaction among primiparous women. Midwifery.

[58] Green JM, Baston HA (2003). Feeling in control during labor: concepts, correlates, and consequences. Birth.

[59] Spiby H, Henderson B, Slade P, Escott D, Fraser RB (1999). Strategies for coping with labour: does antenatal education translate into practice?. J Adv Nurs.

[60] Ho I, Holroyd E (2002). Chinese women’s perceptions of the effectiveness of antenatal education in the preparation for motherhood. J Adv Nurs.

[61] Nolan ML (2009). Information giving and education in pregnancy: a review of qualitative studies. J Perinat Educ.

[62] Aston M, Price S, Monaghan J, Sim M, Hunter A, Little V (2018). Navigating and negotiating information and support: experiences of first-time mothers. J Clin Nurs.

[63] Schaad B, Bourquin C, Panese F, Stiefel F (2017). Revue Médicale Suisse : patients : sujets avant d’être partenaires. Rev Med Suisse.

[64] Nieuwenhuijze M, Low LK (2013). Facilitating women’s choice in maternity care. J Clin Ethics.

[65] Elmir R, Schmied V (2016). A meta-ethnographic synthesis of fathers׳ experiences of complicated births that are potentially traumatic. Midwifery.

[66] Schobinger E, Vanetti M, Ramelet AS, Horsch A (2022). Social support needs of first-time parents in the early-postpartum period: a qualitative study. Front Psychiatry.

